# Lack of significant association between serum inflammatory cytokine profiles and the presence of colorectal adenoma

**DOI:** 10.1186/s12885-015-1115-2

**Published:** 2015-03-14

**Authors:** Curtis J Henry, Rebecca L Sedjo, Andrii Rozhok, Jennifer Salstrom, Dennis Ahnen, Theodore R Levin, Ralph D’Agostino, Steven Haffner, James DeGregori, Tim Byers

**Affiliations:** 1 Department of Biochemistry and Molecular Genetics, University of Colorado Anschutz Medical Campus, 12801 East 17th Avenue, MS 8010, Aurora, CO USA 80045; 2 Integrated Department of Immunology, National Jewish Health and the University of Colorado Anschutz Medical Campus, 1400 Jackson Street, Denver, CO USA 80206; 3 Department of Community and Behavioral Health, Colorado School of Public Health, University of Colorado Anschutz Medical Campus, 13001 East 17th Place, MS F519, Aurora, CO USA 80045; 4 Department of Gastroenterology and Hepatology, University of Colorado Denver, Aurora, CO USA 80045; 5 Kaiser Permanente Division of Research, 2000 Broadway, Oakland, CA USA 94612; 6 Department of Biostatistical Sciences, Section on Biostatistics, Wake Forest School of Medicine, Medical Center Boulevard, Winston-Salem, NC USA 27157; 7 University of Texas Health Science Center, 7703 Floyd Curl Dr, San Antonio, TX USA 78229; 8 Department of Epidemiology, Colorado School of Public Health, University of Colorado Anschutz Medical Campus, 13001 East 17th Place, B119 Building 500, Room W3122, Aurora, CO USA 80045

**Keywords:** Colorectal cancer, Adenomas, Inflammation, Biomarkers, IRAS

## Abstract

**Background:**

Inflammatory cytokines in the colonic microenvironment have been shown to increase with advance colorectal cancer disease state. However, the contribution of inflammatory cytokines to pre-malignant disease, such as the formation of adenomas, is unclear.

**Methods:**

Using the Milliplex® MAP Human Cytokine/ Chemokine Magnetic Bead Panel Immunoassay, serum cytokine and chemokine profiles were assayed among participants without an adenoma (n = 97) and those with an adenoma (n = 97) enrolled in the NCI-funded Insulin Resistance Atherosclerosis Colon Study. The concentrations of interleukin-10 (IL-10), IL-1β, IL-6, IL-17A, IL-2, IL-4, IL-7, IL-12(p70), interferon-γ (IFN-γ), macrophage chemoattractant protein-1 (MCP-1), regulated on activation, normal T cell expressed and secreted (RANTES), tumor necrosis factor-alpha (TNF-α), vascular endothelial growth factor (VEGF), granulocyte macrophage colony-stimulating factor (GM-CSF), and macrophage inflammatory protein-1β (MIP-1β) were determined. Multiple logistic regression analyses were used to evaluate the association between adenoma prevalence and cytokine levels.

**Results:**

The presence of colorectal adenomas was not associated with significant increases in the systemic levels of proinflammatory (TNF-α, IL-6, IL-1β) or T-cell polarizing (IL-12, IL-2, IL-10, IL-4, IL-17, IFN-γ) cytokines. Furthermore, MCP-1 and RANTES levels were equivalent in the serum of study participants with and without adenomas.

**Conclusions:**

These findings suggest colorectal adenoma prevalence may not be associated with significant alterations in systemic inflammation.

## Background

Colorectal cancer (CRC) is the second leading cause of cancer-related deaths in the United States, and the prevalence of CRC is increasing worldwide [1,2]. As with most cancers, CRC results from a combination of genetic and environmental factors [3]. Colorectal adenomas and polyps are an early pre-malignant precursor and usually develop 10 years prior to carcinomas [4,5]. Known risk factors associated with adenomatous polyp development include age, smoking, excessive alcohol consumption, obesity, and chronic inflammatory conditions such as inflammatory bowel disease (IBD) [4-6]. Consequently, inflammation has long been suspected to be a major environmental risk factor in CRC development [7-9]. Other evidence supporting the link between inflammation and CRC development comes from the effect of non-steroidal anti-inflammatory drugs (NSAIDs), particularly aspirin, which has been shown to reduce CRC incidence and disease progression [10-13]. More recently, variations in immune cell populations and changes in the gut microbiome have been linked to CRC induction and progression [3,14-20]. While changes such as alterations of the gut microbiome could potentially be used in risk assessment, currently there are no reliable and easily assessed predictors of pre-malignant disease states apart from detection of adenomas [21].

It is widely accepted that inflammation plays a major role in advanced stage CRC disease progression; however, whether it is associated with the formation of pre-malignant lesions is currently unclear. Evidence pointing towards inflammation promoting disease progression from pre-malignant adenomatous polyps to advance staged CRC is suggested by the observation that plasma levels of tumor necrosis factor-alpha (TNF-α), interferon-gamma (IFN-γ)-induced protein 10, and IL-8 increase in patients during each stage of disease progression [22]. Similar results were observed in a mouse model of ulcerative colitis in which the production of the pro-inflammatory cytokines, chemokines, and M2 macrophages increase as the disease progressed from dysplasia to CRC metastasis [23]. Furthermore, colorectal adenomas were found to be more frequent in patients with high endotoxin levels (which promotes the production of pro-inflammatory cytokines) at the time of colonoscopy [3]; however, systemic (plasma) and local (rectal mucosa) cytokine levels were not significantly altered in individuals with adenomas relative to control populations [3]. The correlation between systemic cytokine levels and adenoma status was also addressed in studies determining the role of anti-inflammatory dietary flavonols in colorectal adenoma incidence and prevention [24,25]. In these studies, high levels of flavonol consumption correlated with decreased serum IL-6 levels and reduced adenoma recurrence [25]; however, overall cytokine profiles were not accurate predictors of flavonol consumption or adenoma incidence [24]. Similarly, another study assaying serum profiles between participants with colorectal adenomas, predisposing conditions such as IBD, and those with advanced-stage colon cancer observed that cytokine profiles could only accurately distinguish active IBD populations from those with CRC whereas the profiles between individuals with adenomas and CRC were not reliable enough to distinguish these two cohorts [7].

Unlike advanced stage CRC, studies linking inflammation to the presence or development of pre-malignant lesions are inconsistent. A potential reason for these inconsistencies could be attributed to the inherent degradation of serum cytokines and chemokines in studies assaying the relationship between disease state and altered systemic inflammation [26,27]. Based on the observations that inflammation is increased with advanced stage CRC, we hypothesized that this would also be the case when colorectal adenomas are detectable. Therefore, the aim of our study was to determine if inflammation was increased in study participants with colorectal adenomas relative to control participants using a novel methodology based on the stability of interleukin-7 (IL-7) in serum samples. This approach significantly reduces the inclusion of degraded samples in our study thus increasing the reliability of the data obtained from these populations.

## Methods

### Study participants

A case-control study was conducted as part of a multi-center, multiethnic, prospective cohort study called the Insulin Resistance Atherosclerosis Study (IRAS), which was originally designed to investigate the association between insulin resistance and atherosclerosis. Details of the design and recruitment have been published previously [28,29]. Briefly, 1,625 participants consisting of men and women of non-Hispanic White, Hispanic, and African American race/ethnicity and various glucose tolerance status (normal, impaired glucose tolerance, and diabetes) were recruited between 1992–1994 from four clinical sites (Los Angeles, CA; Oakland, CA; San Luis Valley, CO; San Antonio, TX). Of the original 1,625 participants, 600 participants received colonoscopies, and biopsy specimens were examined between 2002 and 2004 [29]. Participants were recruited for a colonoscopy if they were ≥50 years of age, able to provide informed consent, able to undergo the colonoscopy procedure, and/or likely to benefit from colorectal screening. Participants with a prior screening were only included if the timing of their subsequent surveillance colonoscopy exam was within the study period. The Internal Review Boards and ethics committees of all participating institutions including the University of Colorado Anschutz Medical Campus (Aurora, CO, USA), Kaiser Permanente (Oakland, CA, USA), the University of Texas Health Science Center (San Antonio, Texas, USA), Wake Forest University School of Medicine (Winston-Salem, NC, USA), and the University of California Los Angeles (Los Angeles, CA, USA) approved this study. Written informed consents were obtained from all study participants prior to any research procedures being performed.

For this study, 97 participants with adenomas were selected and 97 corresponding participants with normal colonoscopies from the same clinical site were included as controls. All participants also needed to have had a stored serum sample, taken at the time of the colonoscopy, available for cytokine analyses.

Blood samples were collected following a 12 hour fast of the participants. Red top tubes were used to collect the stored serum samples used for cytokines. These samples were collected, processed, shipped to a central location, and stored at -70°C. In 2008, all samples were shipped by overnight delivery on dry ice to the University of Colorado Anschutz Medical Campus where they were immediately stored at -80°C until they were analyzed for cytokine concentrations in 2013.

### Participant information

Self-reported demographics, smoking practices, NSAID use within the past 2 weeks, and previous screening history were collected through interviewer-administrated questionnaires. Data were collected at baseline (1992–94), the second visit (1997–1999) and at the colonoscopy visit (2002–2004). Trained staff collected anthropometric measures of heights measured to the nearest 0.01 cm and weights measured to the nearest 0.1 kg. Glucose status was determined using a 2-hour, 75-g oral glucose tolerance test with cutoff values based on World Health Organization criteria [30].

### Cytokine analysis

Cytokine profiles were determined using the Human Cytokine/ Chemokine Magnetic Bead Panel protocol from the Milliplex® Map Kit (Cat. No. HCYTOMAG-60K, Billerica, MA). Briefly, cytokine/chemokine assay plates were washed with wash buffer, sealed, and mixed on an orbital plate shaker for 10 minutes at room temperature. The wash buffer was decanted and the standards, assay buffer, or serum samples were mixed with serum matrix in each well. After the addition of the samples or controls, samples were incubated overnight at 4°C on an orbital shaker with fluorescently-labeled capture antibody-coated beads. After overnight incubation with capture antibodies to detect IFN-γ, IL-10, IL-1β, IL-6, MCP-1 (macrophage chemoattractant protein-1), RANTES (regulated on activation, normal T cell expressed and secreted), TNF-α, VEGF, GM-CSF, IL-17A, IL-2, IL-4, MIP-1β (macrophage inflammatory protein-1β), IL-7, and IL-12(p70), well contents were removed via the washing instructions provided by the protocol. Biotinylated detection antibodies were then added to the wells and incubated with samples for 1 hour at room temperature while shaking. After incubation, well contents were removed as previously described and streptavidin-phycoerythrin was added to each well. The samples were incubated with streptavidin-phycoerythrin for 30 minutes at room temperature. After the incubation period, samples were washed as previously described and resuspended in Sheath Fluid. Plates were run on the Luminex MagPix® machine and data were collected using the Luminex xPONENT® software (v. 4.2). Analysis of the cytokine/ chemokine median fluorescent intensity (MFI) was performed using the Milliplex® Analyst software (v. 5.1). The interassay coefficient of variation for all cytokines tested was 11.93%.

### Quality control

Degradation is an inherent issue with studies designed to analyze cytokines and chemokines from thawed serum or plasma samples [26,27]. To account for this problem and to increase the validity of our study, we omitted samples which contained interleukin-7 (IL-7) levels below the limit of detection. This approach was chosen because IL-7 is a homeostatic cytokine that regulates lymphocyte levels and thus should be detectable in the serum of most samples [31]. Furthermore, recent studies have shown that IL-7 is a stable cytokine that can be reliably detected from thawed serum samples after multiple freeze thaws [26,27]. Based on our approach, one-third of our original samples did not meet the IL-7 positive criteria and were therefore not included in the final cytokine analysis due to potential degradation issues which would confound the interpretation of the results (data not shown).

### Statistical analysis

The outcome variable of adenoma status was determined by the highest grade lesion identified. Those participants classified as “any adenoma” included participants with an advanced adenomatous polyp (n = 31) defined those polyps with villous or mixed tubulovillous features with high-grade dysplasia or >1 cm in diameter and those participants with a non-advanced adenomatous polyp (n = 66) defined as tubular histology under 1 cm in diameter. None of the polyps had carcinoma. Information was not collected on whether the polyps were serrated. Control participants without an adenoma had no polyps, including hyperplastic polyps.

Cytokines were categorized into three groups (low, medium, and high) based on the distributions among those participants without any adenomas. For ten of the cytokines (GM-CSF, IFN-γ, IL-10, IL-12p70, IL-17A, IL-1β, IL-2, IL-4, IL-6, and VEGF), the lowest category included those participants with a cytokine level below detectable levels. The other two categories were determined based on the cutoff values of the median in the control group. For the MCP-1, MIP-1β, TNF-α, IL-7, and RANTES, there were no (RANTES) or a minimal number of serum samples with levels of these cytokines below the detectable level (MCP-1, n = 1; and MIP-1β, n = 4); therefore, tertiles were generated for each cytokine based on their distribution in the control group. Based on standard criteria, body mass index (BMI) was calculated (kg/m^2^) and categorized as normal (<25 kg/m^2^), overweight (25 to >30 kg/m^2^), and obese (≥30 kg/m^2^) [29].

Chi-squared tests were used to evaluate differences between participants with and without an adenoma by categorical variables of study center, sex, age, race/ethnicity, glucose tolerance status at the first visit, BMI at colonoscopy visit, smoking status, previous screening, NSAID use within the previous 2 weeks of the colonoscopy visit. Variables that were significant at p ≤ 0.10 in the bivariate analysis were included in the multivariate analyses which included age, sex, and previous screening. Multivariate unconditional logistic regression was used to evaluate the association between adenoma prevalence and the categories of each cytokine individually while controlling for potential confounders. Unadjusted and adjusted odds ratios and 95% confidence intervals (CI) were estimated. Linear trends were calculated by treating the categorical variable as continuous in the logistic models. Intercooled Stata version 11 (StataCorp, *Stata Statistical Software: Release 11,* College Station, TX, StataCorp LP, 2009) was used to conduct all analyses based on 2-sided statistical tests with an alpha level of 0.05.

Pair-wise correlations between cytokine levels were done by either Pearson or Spearman correlation coefficients, depending on normality of distributions of each cytokine samples measured by D’Agostino’s K^2^ test [32,33]. The parametric Pearson method was only used in cases when both samples in the compared pair were normally distributed. Otherwise, the samples were compared by a Spearman non-parametric correlation test. Correlations were called significant using the 0.05 *p*-value threshold. Non-significant correlations are not presented.

## Results

### Epidemiologic results

Among the 194 IRAS Colon Study participants included in this analysis, older men who had previously reported being screened for colorectal cancer were significantly associated with adenoma prevalence in the bivariate analysis (Table 1). No significant associations were observed between adenoma status and center, race/ethnicity, glucose tolerance status, BMI, NSAID use, and smoking status. The participants in our study did not report having predisposing pro-inflammatory conditions such as IBD or chronic infections; however, we observed that factors known to be associated with elevated inflammation (advanced age; [22,34]) were significantly associated with the presence of adenomas (Table 1).

**Table 1:** **Prevalence of colorectal adenoma by demographic characteristic and risk factors among a nested case-control of IRAS Colon study participants**

Characteristic	No Adenoma N = 97	Any Adenoma N = 97	p-value
Center			
San Antonio, TX	26 (50.0)	26 (50.0)	1.00
San Luis Valley, CO	20 (50.0)	20 (50.0)	
Oakland, CA	34 (50.0)	34 (50.0)	
Los Angeles, CA	17 (50.0)	17 (50.0)	
Sex			
Male	33 (39.8)	50 (60.2)	0.01
Female	64 (57.7)	47 (42.3)	
Age			
<60	36 (62.1)	22 (37.9)	<0.01
60-69	42 (53.9)	36 (46.2)	
70+	19 (32.8)	39 (67.2)	
Race/Ethnicity			
Non-Hispanic White	32 (43.2)	42 (56.8)	0.14
Black	35 (60.3)	23 (39.7)	
Hispanic	30 (48.4)	32 (51.6)	
Glucose tolerance at 1992-94			
Normoglucose	54 (49.5)	55 (50.5)	0.68
Impaired glucose tolerance	20 (57.1)	15 (42.9)	
Type II diabetes mellitus	23 (47.9)	25 (52.1)	
BMI (kg/m^2^) at 2002-2004			
Normal (<25kg/m^2^)	54 (50.0)	54 (50.0)	1.00
Overweight (25-29.9 kg/m^2^)	19 (50.0)	19 (50.0)	
Obese (30 + kg/m^2^)	24 (50.0)	24 (50.0)	
Smoking at 2002-2004			
Never	51 (53.7)	44 (46.3)	0.32
Ever	46 (46.7)	53 (53.5)	
Self reported previous screening			
None	34 (54.8)	28 (45.2)	<0.01
Yes, without polyp	56 (54.9)	46 (45.1)	
Yes, with polyp	7 (23.3)	23 (76.7)	
NSAID use			
None or Don’t Know	33 (45.2)	40 (54.8)	0.30
Yes	64 (52.9)	57 (47.1)	

Population data for participants included in the Insulin Resistance Atherosclerosis Study (IRAS). Details of the design and recruitment have been published previously [29]. Numbers in the parentheses represent the percentage of the total individuals included in each category. Statistical significance was determined used Chi-squared test.

### Normal biological relationships are observed in serum cytokines used for analysis

**Figure 1:**
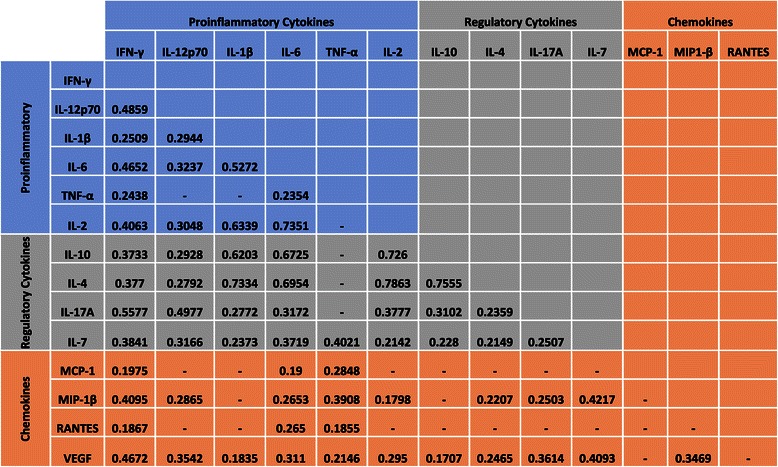
**Correlation between cytokines present in the serum of study participants.** Spearman or Pearson correlation criteria were used in each pairwise comparison based on the distributions of values in each cytokine pair at the significance level of 0.05; only significantly correlated values are presented. Colourwash panels show intensity of FDG uptake and PF.

Pairwise correlation analysis was used to determine if the expected relationships existed between cytokines assayed in this study in the IL-7 positive samples, which were used to assay if elevated cytokine profiles were observed in participants with adenomas relative to those without an adenoma. Interferon gamma, which is a potent inducer of chemokines [35], tracked significantly with chemokines such as MIP-1β (0.4095, Figure 1). The cytokine IL-12p70 is a potent inducer of IFN-γ production from T-lymphocytes [36] and antagonizes the actions of the anti-inflammatory cytokine IL-10 [37]. A modest positive correlation was observed between IL-12p70 and IL-10 (0.2928, Figure 1), and a stronger correlation was observed between IL-12p70 and IFN-γ (0.4895, Figure 1). The hallmark proinflammatory cytokines IL-6 and IL-1β also significantly tracked together (0.5272, Figure 1). Furthermore, the Th1-associated cytokine TNF-α did not track with the Th2-skewing cytokine IL-4 (Figure 1). Based on the existing correlations between the cytokines in our serum samples, we determined if the increased inflammation was associated with the presence of adenomas.

### Adenomas are not associated with altered serum cytokine levels

**Figure 2:**
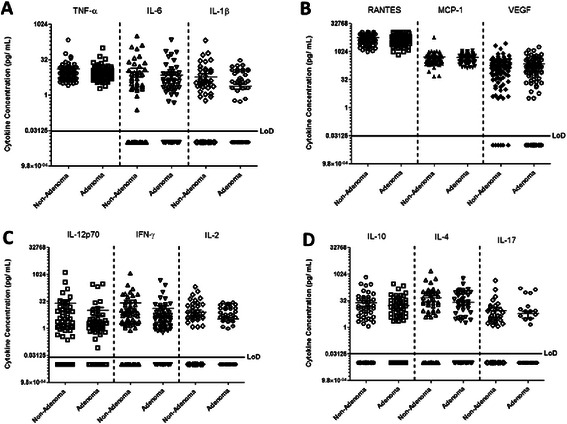
**Healthy patients and those with adenomas exhibit similar serum cytokine profiles.** Serum samples from healthy individuals and those with adenomas were subjected to cytokine analysis using the Human Cytokine/ Chemokine Magnetic Bead Panel protocol from Milliplex®. Shown are the concentrations of selected proinflammatory cytokines **(A)**, chemokines **(B)**, cytokines that activate T-cell responses **(C)**, and those that regulate T-cell polarization/ differentiation **(D)**. The levels of IL-7 in all of the analyzed samples were not significantly different between non-adenoma and adenoma positive study participants (data not shown). MIP-1β and GM-CSF were undetectable in most samples and found only at low levels in samples that were included in the cytokine analysis (data not shown).

Analyses of the proinflammatory cytokines TNF-α, IL-6, and IL-1β in the serum of participants with colorectal adenomas compared to non-adenoma controls (Figure 2A) revealed that the presence of adenomas do not lead to systemic elevation in the levels of these cytokines. In addition, we observed similar serum chemokine levels in participants with adenomas present at the time of the colonoscopy relative to participants without detectable adenomas (Figure 2B). Furthermore, cytokines that are known to promote (IL-12, IFN-γ, and IL-2) or regulate (IL-10, IL-4, and IL-17) T-cell polarization and differentiation were similar in the serum of individuals with and without detectable adenomas (Figure 2C and D).

Although serum cytokines levels were not significantly increased in participants with adenomas relative to non-adenoma controls, we observed that the average concentrations for the T-cell activating cytokines IL-12p70 (non-adenoma mean of 24.05 pg/mL; adenoma mean of 10.16 pg/mL), IFN-γ (non-adenoma mean of 29.28 pg/mL; adenoma mean of 15.13 pg/mL), and IL-2 (non-adenoma mean of 7.44 pg/mL; adenoma mean of 3.56 pg/mL), while not significantly different, were lower in patients with adenomas relative to control subjects. Furthermore, when we adjusted the data for age, sex, and previous screening, we observed that the levels of the chemokine RANTES was significantly lower in patients with adenomas relative to controls (0.04, Table 2). These data indicate that minor changes in systemic inflammatory status may be associated with colorectal adenomas, but the robust induction of cytokines is not associated with the development of these conditions. These results were confirmed in the logistic regression analysis which revealed that no association existed between colorectal adenomas and GM-CSF, IFN-γ, IL-10, IL-12p70, IL-17A, IL-1β, IL-2, IL-4, IL-6, IL-7, MCP-1, MIP-1β, and VEGF after adjustment for age, sex, and previous colorectal screening (Table 2).

**Table 2:** **Associations between cytokines and colorectal adenomas among participants in the IRAS Colon Study**

Cytokines	Adenoma prevalence n/N (%)	Crude OR (95% CI)	Adjusted OR (95% CI)
**GM-CSF**			
**L**	67/124 (54.0)	1.00	1.00
**M**	17/37 (46.0)	0.72 (0.35 1.51)	0.83 (0.38 1.82)
**H**	13/33 (39.4)	0.55 (0.25 1.21)	0.70 (0.30 1.62)
**p for trend**			0.38
**Interferon gamma**			
**L**	37/77 (48.1)	1.00	1.00
**M**	42/71 (59.2)	1.57 (0.82 3.00)	1.35 (0.67 2.72)
**H**	18/46 (39.1)	0.69 (0.33 1.46)	0.77 (0.35 1.69)
**p for trend**			0.67
**IL-10**			
**L**	55/112 (49.1)	1.00	1.00
**M**	18/38 (47.4)	0.93 (0.45 1.95)	0.91 (0.41 2.00)
**H**	24/44 (54.6)	1.24 (0.62 2.50)	1.45 (0.69 3.08)
**p for trend**			0.40
**IL-12p70**			
**L**	50/103 (48.5)	1.00	1.00
**M**	35/57 (61.4)	1.69 (0.87 3.26)	1.86 (0.92 3.76)
**H**	12/34 (35.3)	0.58 (0.26 1.29)	0.59 (0.25 1.42)
**p for trend**			0.68
**IL-17A**			
**L**	82/152 (54.0)	1.00	1.00
**M**	5/19 (26.3)	0.30 (0.10 0.89)	0.42 (0.14 1.30)
**H**	10.23 (43.5)	0.66 (0.27 1.59)	0.83 (0.33 2.13)
**p for trend**			0.40
**IL-18**			
**L**	67/127 (52.8)	1.00	1.00
**M**	12/31 (38.7)	0.57 (0.25 1.26)	0.81 (0.34 1.96)
**H**	18/36 (50.0)	0.90 (0.43 1.88)	1.09 (0.50 2.41)
**p for trend**			0.93
**IL-2**			
**L**	37/132 (50.8)	1.00	1.00
**M**	15/31 (48.4)	0.91 (0.42 1.99)	1.25 (0.53 2.96)
**H**	15/31 (48.4)	0.91 (0.42 1.99)	1.14 (0.49 2.62)
**p for trend**			0.67
**IL-4**			
**L**	56/117 (47.9)	1.00	1.00
**M**	24/42 (57.1)	1.45 (0.71 2.96)	1.83 (0.84 3.98)
**H**	17/35 (48.6)	1.03 (0.48 2.19)	1.33 (0.59 3.02)
**p for trend**			0.29
**IL-6**			
**L**	53/117 (45.3)	1.00	1.00
**M**	29/46 (63.0)	2.06 (1.02 4.15)	2.57 (1.19 5.54)
**H**	15/31 (48.4)	1.13 (0.51 2.50)	1.35 (0.58 3.19)
**p for trend**			0.17
**IL-7**			
**L**	40/73 (54.8)	1.00	1.00
**M**	28/60 (46.7)	0.72 (0.36-1.43)	0.59 (0.28-1.24)
**H**	29/61 (47.5)	0.75 (0.38-1.48)	0.60
**p for trend**			
**MIP-1β**			
**L**	22/53 (41.5	1.00	1.00
**M**	53/87 (60.9)	2.20 (1.10-4.40	2.69 (1.25-5.80)
**H**	22/54 (4.7)	0.97 (0.45-2.09)	1.25 (0.54-2.91)
**p for trend**			0.65
**RANTES**			
**L**	46/79 (58.2)	1.00	1.00
**M**	32/64 (50.0)	0.72 (0.37-1.39)	0.66 (0.33-1.35)
**H**	19/51 (37.3)	0.43 (0.21-0.88)	0.46 (0.21-0.99)
**p for trend**			0.04
**VEGF**			
**L**	13/19 (68.4)	1.00	1.00
**M**	40/86 (46.5)	0.40 (0.14-1.15)	0.42 (0.14-1.28)
**H**	44/89 (49.4)	0.45 (0.46-1.29)	0.62 (0.20-1.88)
**p for trend**			0.97
**TNFα**			
**L**	31/64 (48.9)	1.00	1.00
**M**	37/68 (54.4)	1.27 (0.64-2.51)	1.27 (0.64-2.52)
**H**	29/62 (46.8)	0.93 (0.46-1.88)	1.14 (0.55-2.35)
**p for trend**			0.54

Tertiles were generated with those undetectable as the referent value and then the other tertiles based on the median of cytokines values from participants with normal colonoscopies except for those in bold. Those in bold had no or only a few undetectable values so tertiles were based on the distribution in normal point. Multivariate unconditional logistic regression was used to evaluate the association between adenoma prevalence and the categories of each cytokine individually while controlling for potential confounders. Low (denoted by L), medium (denoted by M), and high (denoted by H) cytokine concentrations are indicated. Statistically significant results indicated in bold type.

## Discussion

Whether inflammation causes CRC or is induced by CRC is still an area of heavy investigation; however, what is well established is that inflammation increases as disease progresses [18,19,23]. Unlike the clear correlation that exists between advanced-stage CRC development and inflammation, the role of inflammatory conditions in promoting the formation of high-risk conditions such as adenomas is unclear [3,7,38]. Our results did not find a correlation between adenoma status and alterations in circulating cytokine and chemokine levels, which is consistent with other reports showing that the presence of adenomas do not alter systemic cytokine and chemokine profiles [3,7,24].

Although we did not observe significant alterations in serum cytokine levels in study participants with adenomas relative to controls, we did observe an overall decrease in the serum levels of IL-2, IL-12p70, and IFN-γ in participants with adenomas as compared to those without an adenoma. In the Bobe *et al.* study, the presence of adenomas correlated with reduced serum IL-2 levels which was associated with an increase in adenoma recurrence [24]. Furthermore, NSAIDs usage correlates with reduced adenoma and CRC incidence [7,39,40]. Moreover, once pre-malignant lesions are detected, NSAIDs and flavonols have demonstrated protective effects against the transition from colorectal adenomas to advanced stage CRC [25,39,41-43] which are thought to be mediated largely by their anti-inflammatory properties [43]. These results indicate that inflammation may play a role in the development adenomas; however, our results and others suggest that the development of pre-malignant stages of disease is not associated with substantial changes in systemic inflammation.

One limitation of this study is due to the large amount of samples that were below the detectable level of the assay which lead to a relatively small sample size thus reducing the power of the study to detect statistically significant associations. However, this study had at least 80% power to detect an odds ratio of 0.17 or 2.75. Furthermore, by utilizing only those samples from participants in which IL-7 could be reliability measured, we are confident that degradation was not an issue with the included samples. We assume that the removal of degraded samples would be non-differential and therefore not bias the outcome of the study.

## Conclusion

By using a stringent quality control method to exclude the analysis of potentially degraded samples, we have concluded that there is no large association between colorectal adenomas status and systemic levels of proinflammatory (TNF-α, IL-6, IL-1β) or T-cell polarizing (IL-12, IL-2, IL-10, IL-4, IL-17, IFN-γ) cytokines among a subset of participants from the IRAS Colon Study. Furthermore, no association between adenoma status and alterations in systemic chemokines (RANTES, MCP-1) were observed in this study. Our observations are consistent with other studies, further suggesting that the induction of robust systemic inflammation is not associated with the presence of detectable colorectal adenomas; however, small associations are possible. In these studies we did not address the association between adenoma status and local alterations in the colonic microenvironment; therefore, changes in local inflammation and alterations of the gut microbiome should be explored in future studies.

## Abbreviations

CRC: Colorectal cancer
IL: Interleukin
TNF: Tumor necrosis factor
IFN: Interferon
IBD: Inflammatory bowel disease
NSAIDS: Non-steroidal anti-inflammatory drugs
MCP-1: Macrophage chemoattractant protein-1
RANTES: Regulated on activation, normal T cell expressed and secreted
VEGF: Vascular endothelial growth factor
GMCSF: Granulocyte macrophage colony-stimulating factor
MIP-1β: Macrophage inflammatory protein-1β
IRAS: Insulin Resistance Atherosclerosis Study

## References

[1] Jemal A, Siegel R, Ward E, Hao Y, Xu J, Thun MJ (2009). Cancer statistics, 2009. CA Cancer J Clin.

[2] Jemal A, Bray F, Center MM, Ferlay J, Ward E, Forman D (2011). Global cancer statistics. CA Cancer J Clin.

[3] Kang M, Edmundson P, Araujo-Perez F, McCoy AN, Galanko J, Keku TO (2013). Association of plasma endotoxin, inflammatory cytokines and risk of colorectal adenomas. BMC Cancer.

[4] Olsen HW, Lawrence WA, Snook CW, Mutch WM (1988). Risk factors and screening techniques in 500 patients with benign and malignant colon polyps. An urban community experience. Dis Colon Rectum.

[5] Atkin WS, Saunders BP (2002). Surveillance guidelines after removal of colorectal adenomatous polyps. Gut.

[6] Terzic J, Grivennikov S, Karin E, Karin M (2010). Inflammation and colon cancer. Gastroenterology.

[7] Krzystek-Korpacka M, Diakowska D, Kapturkiewicz B, Bebenek M, Gamian A (2013). Profiles of circulating inflammatory cytokines in colorectal cancer (CRC), high cancer risk conditions, and health are distinct. Possible implications for CRC screening and surveillance. Cancer Lett.

[8] Lakatos L, Mester G, Erdelyi Z, David G, Pandur T, Balogh M (2006). Risk factors for ulcerative colitis-associated colorectal cancer in a Hungarian cohort of patients with ulcerative colitis: results of a population-based study. Inflamm Bowel Dis.

[9] Lee WS, Baek JH, You DH, Nam MJ (2013). Prognostic value of circulating cytokines for stage III colon cancer. J Surg Res.

[10] Wang D, Dubois RN (2010). Eicosanoids and cancer. Nat Rev Cancer.

[11] Waddell WR, Loughry RW (1983). Sulindac for polyposis of the colon. J Surg Oncol.

[12] Labayle D, Fischer D, Vielh P, Drouhin F, Pariente A, Bories C (1991). Sulindac causes regression of rectal polyps in familial adenomatous polyposis. Gastroenterology.

[13] Giardiello FM, Hamilton SR, Krush AJ, Piantadosi S, Hylind LM, Celano P (1993). Treatment of colonic and rectal adenomas with sulindac in familial adenomatous polyposis. N Engl J Med.

[14] Sobhani I, Tap J, Roudot-Thoraval F, Roperch JP, Letulle S, Langella P (2011). Microbial dysbiosis in colorectal cancer (CRC) patients. PLoS ONE.

[15] Kostic AD, Gevers D, Pedamallu CS, Michaud M, Duke F, Earl AM (2012). Genomic analysis identifies association of Fusobacterium with colorectal carcinoma. Genome Res.

[16] Scanlan PD, Shanahan F, Clune Y, Collins JK, O'Sullivan GC, O'Riordan M (2008). Culture-independent analysis of the gut microbiota in colorectal cancer and polyposis. Environ Microbiol.

[17] Wang T, Cai G, Qiu Y, Fei N, Zhang M, Pang X (2012). Structural segregation of gut microbiota between colorectal cancer patients and healthy volunteers. Isme J.

[18] Guffey CR, Fan D, Singh UP, Murphy EA (2013). Linking obesity to colorectal cancer: recent insights into plausible biological mechanisms. Curr Opin Clin Nutr Metab Care.

[19] Huang X, Zou Y, Lian L, Wu X, He X, Huang Y (2013). Changes of T Cells and Cytokines TGF-beta1 and IL-10 in Mice during Liver Metastasis of Colon Carcinoma: Implications for Liver Anti-tumor Immunity. J Gastrointest Surg.

[20] Liu Z, Brooks RS, Ciappio ED, Kim SJ, Crott JW, Bennett G (2012). Diet-induced obesity elevates colonic TNF-alpha in mice and is accompanied by an activation of Wnt signaling: a mechanism for obesity-associated colorectal cancer. J Nutr Biochem.

[21] Tanaka T, Tanaka M, Tanaka T, Ishigamori R (2010). Biomarkers for colorectal cancer. Int J Mol Sci.

[22] Choi JW, Liu H, Shin DH, Yu GI, Hwang JS, Kim ES (2013). Proteomic and cytokine plasma biomarkers for predicting progression from colorectal adenoma to carcinoma in human patients. Proteomics.

[23] Wang W, Li X, Zheng D, Zhang D, Huang S, Zhang X (2013). Dynamic changes of peritoneal macrophages and subpopulations during ulcerative colitis to metastasis of colorectal carcinoma in a mouse model. Inflamm Res.

[24] Bobe G, Murphy G, Albert PS, Sansbury LB, Lanza E, Schatzkin A (2010). Serum cytokine concentrations, flavonol intake and colorectal adenoma recurrence in the Polyp Prevention Trial. Br J Cancer.

[25] Bobe G, Albert PS, Sansbury LB, Lanza E, Schatzkin A, Colburn NH (2010). Interleukin-6 as a potential indicator for prevention of high-risk adenoma recurrence by dietary flavonols in the polyp prevention trial. Cancer Prev Res (Phila).

[26] Chaturvedi AK, Kemp TJ, Pfeiffer RM, Biancotto A, Williams M, Munuo S (2011). Evaluation of multiplexed cytokine and inflammation marker measurements: a methodologic study. Cancer Epidemiol Biomarkers Prev.

[27] Parkitny L, McAuley JH, Kelly PJ, Di Pietro F, Cameron B, Moseley GL (2013). Multiplex cytokine concentration measurement: how much do the medium and handling matter?. Mediators Inflamm.

[28] Wagenknecht LE, Mayer EJ, Rewers M, Haffner S, Selby J, Borok GM (1995). The insulin resistance atherosclerosis study (IRAS) objectives, design, and recruitment results. Ann Epidemiol.

[29] Sedjo RL, Byers T, Levin TR, Haffner SM, Saad MF, Tooze JA (2007). Change in body size and the risk of colorectal adenomas. Cancer Epidemiol Biomarkers Prev.

[30] Diabetes mellitus (1985). Report of a WHO Study Group. World Health Organ Tech Rep Ser.

[31] Lee SK, Surh CD (2005). Role of interleukin-7 in bone and T-cell homeostasis. Immunol Rev.

[32] D’Agostiono RB (1970). Transformation to normality of the null distribution of *g*_*1*_. Biometrika.

[33] D’Agostino RB, Belanger A, D’Agostino RB (1990). A suggestion for using powerful and informative tests of normality. Am Stat.

[34] Navarrete-Reyes AP, Montana-Alvarez M (2009). Inflammaging, Aging inflammatory origin. Rev Invest Clin.

[35] Rotondi M, Chiovato L (2011). The chemokine system as a therapeutic target in autoimmune thyroid diseases: a focus on the interferon-gamma inducible chemokines and their receptor. Curr Pharm Des.

[36] Trinchieri G (2003). Interleukin-12 and the regulation of innate resistance and adaptive immunity. Nat Rev Immunol.

[37] O'Garra A, Murphy KM (2009). From IL-10 to IL-12: how pathogens and their products stimulate APCs to induce T(H)1 development. Nat Immunol.

[38] Kim S, Keku TO, Martin C, Galanko J, Woosley JT, Schroeder JC (2008). Circulating levels of inflammatory cytokines and risk of colorectal adenomas. Cancer Res.

[39] Half E, Arber N (2009). Colon cancer: preventive agents and the present status of chemoprevention. Expert Opin Pharmacother.

[40] Huang WK, Chiou MJ, Yu KH, Lin YC, Yang TS, Chen JS (2013). The association between low-dose aspirin use and the incidence of colorectal cancer: a nationwide cohort study. Aliment Pharmacol Ther.

[41] Chan AT, Arber N, Burn J, Chia WK, Elwood P, Hull MA (2012). Aspirin in the chemoprevention of colorectal neoplasia: an overview. Cancer Prev Res (Phila).

[42] Chia WK, Ali R, Toh HC (2012). Aspirin as adjuvant therapy for colorectal cancer--reinterpreting paradigms. Nat Rev Clin Oncol.

[43] Wang D, DuBois RN (2013). The role of anti-inflammatory drugs in colorectal cancer. Annu Rev Med.

